# Statistically optimized biotransformation protocol for continuous production of L-DOPA using *Mucuna monosperma* callus culture

**DOI:** 10.1186/2193-1801-2-570

**Published:** 2013-10-28

**Authors:** Shrirang Appasaheb Inamdar, Shripad Nagnath Surwase, Shekhar Bhagwan Jadhav, Vishwas Anant Bapat, Jyoti Prafull Jadhav

**Affiliations:** Department of Biotechnology, Shivaji University, Kolhapur, 416 004 India; Department of Biochemistry, Shivaji University, Kolhapur, 416 004 India; Department of Microbiology, Shivaji University, Kolhapur, 416 004 India

**Keywords:** Biotransformation, Continuous culture, L-DOPA, *Mucuna monosperma*, Response surface methodology

## Abstract

L-DOPA (3,4-dihydroxyphenyl-L-alanine), a modified amino acid, is an expansively used drug for the Parkinson’s disease treatment. In the present study, optimization of nutritional parameters influencing L-DOPA production was attempted using the response surface methodology (RSM) from *Mucuna monosperma* callus. Optimization of the four factors was carried out using the Box–Behnken design. The optimized levels of factors predicted by the model include tyrosine 0.894 g l^-1^, pH 4.99, ascorbic acid 31.62 mg l^-1^and copper sulphate 23.92 mg l^-1^, which resulted in highest L-DOPA yield of 0.309 g l^-1^. The optimization of medium using RSM resulted in a 3.45-fold increase in the yield of L-DOPA. The ANOVA analysis showed a significant *R*^2^ value (0.9912), model *F*-value (112.465) and probability (0.0001), with insignificant lack of fit. Optimized medium was used in the laboratory scale column reactor for continuous production of L-DOPA. Uninterrupted flow column exhibited maximum L-DOPA production rate of 200 mg L^-1^ h^-1^ which is one of the highest values ever reported using plant as a biotransformation source. L-DOPA production was confirmed by HPTLC and HPLC analysis. This study demonstrates the synthesis of L- DOPA using *Mucuna monosperma* callus using a laboratory scale column reactor.

## Background

Parkinson’s disease (PD) is a progressive disorder of the nervous system primarily affecting the motor system of the body and is also known as “Shaking palsy”. PD is the second most common neurodegenerative disorder and the most common movement disorder. The most effective therapy for PD is administration of a modified amino acid known as L-DOPA (3-(3, 4-dihydroxyphenyl)-L-alanine), which is converted to dopamine in the brain. L-DOPA, a dopamine precursor, either alone or in combination with an aromatic amino acid decarboxylase inhibitor (carbidopa, benserazide) is the most effective drug for the treatment of PD, since dopamine fails to pass through the blood brain barrier (Kofman 1971). L-DOPA is marketed as tablets under various brand names, of Sinemet®, Atamet®, Parcopa®, and Stalevo® (Ali and Haq 2006). The world market for L-DOPA is about 250 t/year, and the total market volume is about $101 billion per year (Koyanagi et al. 2005). The natural source consisting of seeds of *M. pruriens* and allied species are widely used for medication since chemical synthesis of this drug is costly and hindered with disadvantage of racemic mixture which inhibits the dopa decaboxylase activity in human body (Krishnaveni et al. 2009).

The bacterial and fungal sources that have been reported earlier for the production of L-DOPA include *Erwinia herbicola* (Koyanagi et al., 2005), *Aspergillus oryzae* (Ali and Haq 2006), *Yarrowia lipolytica* (Ali et al. 2007), *Acremonium rutilum* (Krishnaveni et al. 2009), and *Bacillus* sp. JPJ (Surwase and Jadhav 2011). However, plant sources such as seeds of *Mucuna pruriens* (Chattopadhyay et al. 1994), and *Mucuna monosperma* (Inamdar et al. 2012) are found to be the rich sources of this drug. Cell suspension cultures of *Mucuna pruriens* (Chattopadhyay et al. 1994), banana (Bapat et al. 2000) and *Portulaca grandiflora* (Rani et al. 2007) have also been used for L-DOPA production.

The higher production cost using chemical synthesis and higher commercial value of L-DOPA necessitated to explore the additional sources as well as methods, which would result in maximum production. In this connection, feasibility of cell cultures for metabolite synthesis has been widely reported. However, the optimal design of the culture medium is a very important and indispensable parameter in the scale up of the product based on fermentation processes. The conventional method for medium optimization at bench level involves changing one parameter at a time while keeping all others constant which may be very expensive and time-consuming. In addition, it fails to determine the combined effect of different factors (Lee et al. 2003; Zhang et al. 2007). Statistical experimental designs have been used to address these problems, such as the response surface methodology (RSM).

In the present investigation, callus cultures of *M. monosperma*, was established and used as an efficient alternate source for biotransformation of L-tyrosine to L-DOPA. Critical process parameters were screened initially by one factor at a time, while optimization of these factors was carried out using response surface methodology with a Box-Behnken design.

## Results and discussion

L-DOPA, a drug of choice for Parkinson’s disease, has been reported from cell and suspension cultures of *Mucuna pruriens* (Chattopadhyay et al. 1994, *Musa sps.* (Bapat et al. 2000) and *Portulaca grandiflora* (Rani et al. 2007). However, less or no efforts were attempted towards development of a cost effective process for L-DOPA production using plant sources by biotransformation approach.

*Mucuna monosperma* seeds are a good source of L-DOPA and possess tyrosinase activity (Inamdar et al. 2012). Hence, callus cultures were established using endosperm as explants for detecting presence of L- DOPA *in vitro* for further studies. Medium supplemented with NAA (1.0 mg L^-1^) and 2, 4-D (1.0 mg L^-1^) glutamine (500 mg L^-1^) was used for callus initiation and proliferation.

### Medium optimization by response surface methodology

Medium optimization using the Box-Behnken design was carried out with the components found to be significant from earlier experiments and literature, which include L-tyrosine (A), pH (B), ascorbic acid (C), and CuSO_4_ (D). Table 1 presents the design matrix and the results of the 29 experiments carried out using the Box-Behnken design consisting of 24 trials plus 5-centre points. The results obtained were analyzed by ANOVA using the Design expert software (version 8.0, Stat-Ease Inc. USA), and the regression model was given as:

**Table 1:** **The Box–Behnken design matrix for coded variables along with actual and predicted responses for L-DOPA production**

Std order	L-tyrosine	pH	Ascorbic acid	Copper sulphate	Actual value	Predicted value	Externally studentized residual
1	-1	-1	0	0	0.082525	0.073007	1.524624
2	1	-1	0	0	0.152542	0.146935	0.849911
3	-1	1	0	0	0.164582	0.156311	1.296822
4	1	1	0	0	0.297604	0.293243	0.653778
5	0	0	-1	-1	0.193922	0.190677	0.483016
6	0	0	1	-1	0.247224	0.241344	0.893731
7	0	0	-1	1	0.19848	0.190482	1.24883
8	0	0	1	1	0.182934	0.172301	1.742365
9	-1	0	0	-1	0.185856	0.186702	-0.12492
10	1	0	0	-1	0.297838	0.30429	-0.98693
11	-1	0	0	1	0.164582	0.16424	0.050484
12	1	0	0	1	0.25225	0.257514	-0.79525
13	0	-1	-1	0	0.056224	0.06576	-1.52784
14	0	1	-1	0	0.2	0.193774	0.949833
15	0	-1	1	0	0.082876	0.095211	-2.1082
16	0	1	1	0	0.200234	0.196809	0.510363
17	-1	0	-1	0	0.15	0.158725	-1.37812
18	1	0	-1	0	0.235885	0.235094	0.116827
19	-1	0	1	0	0.137347	0.145906	-1.34827
20	1	0	1	0	0.281356	0.280399	0.141305
21	0	-1	0	-1	0.140269	0.133925	0.969141
22	0	1	0	-1	0.214611	0.222782	-1.27897
23	0	-1	0	1	0.073758	0.073356	0.059289
24	0	1	0	1	0.2	0.214112	-2.55016
25	0	0	0	0	0.294	0.3012	-0.78455
26	0	0	0	0	0.306	0.3012	0.516286
27	0	0	0	0	0.31	0.3012	0.970304
28	0	0	0	0	0.296	0.3012	-0.56031
29	0	0	0	0	0.3	0.3012	-0.12785

(-1) low level, (+1) high level, (0) center point.

L-DOPA = +0.30 + 0.053 × A +0.057 × B + 8.121E-003 × C −0.017 × D +0.016 × A × B + 0.015 × A × C - 6.078E + 0.013 × B × D −0.017 × C × D - 0.033 × A^2^ - 0.10 × B^2^ - 0.063 × C^2^ -0.040 × D^2^. (Equation 1)

Where A is L-tyrosine, B is pH, C is ascorbic acid and D is CuSO_4._

ANOVA of regression model demonstrates that the model is highly significant, as it is evident from the Fisher’s *F*-test with a very low probability value [(Pmodel > *F*) = 0.0001]. The model F value of 112.465 implies that the model is significant. There was only a 0.01% chance that a model F value this large could occur due to noise. Determination coefficient (*R*^2^) was used to check the goodness of fit of the model. In this case, the value of the determination coefficient was *R*^2^ = 0.9912. The value of the adjusted determination coefficient (Adj *R*^2^ = 0.9824) was in reasonable agreement with the Pred R^2^ (0.9539). The lack-of-fit value for regression Eq. (1) was not significant (0.1690), indicating that the model equation was adequate for predicting the L-DOPA production under any combination of values of the variables. "Adeq Precision" measures the signal-to-noise ratio, with a ratio greater than 4 considered as desirable (Anderson and Whitcomb 2005). The "Adeq Precision" ratio of 32.77 obtained in this study indicates an adequate signal. Thus, this model can be used to navigate the design space (Table 2).

**Table 2:** **Analysis of variance (ANOVA) for the fitted quadratic polynomial model of L-DOPA production**

Source	Sum of squares	df	Mean square	F value	p-value prob > F
**Model**	0.161208	14	0.011515	112.4651	< 0.0001
**A-tyrosine**	0.033347	1	0.033347	325.6965	< 0.0001
**B-pH**	0.039541	1	0.039541	386.1985	< 0.0001
**C-ascorbic acid**	0.000791	1	0.000791	7.730531	0.0147
**D-copper sulphate**	0.003595	1	0.003595	35.1166	< 0.0001
**AB**	0.000992	1	0.000992	9.692514	0.0076
**AC**	0.000845	1	0.000845	8.249136	0.0123
**AD**	0.000148	1	0.000148	1.443396	0.2495
**BC**	0.000174	1	0.000174	1.704024	0.2128
**BD**	0.000673	1	0.000673	6.576955	0.0225
**CD**	0.001185	1	0.001185	11.57416	0.0043
**A** ^**2**^	0.007211	1	0.007211	70.42724	< 0.0001
**B** ^**2**^	0.065495	1	0.065495	639.6834	< 0.0001
**C**_**2**_	0.025604	1	0.025604	250.0717	< 0.0001
**D** ^**2**^	0.010209	1	0.010209	99.7092	< 0.0001
**Residual**	0.001433	14	0.000102		
**Lack of fit**	0.001253	10	0.000125	2.771251	0.1690

### Response surface curves

Response surface plots elucidate the relationship between response and experimental level of each variable in presence of other variables at different experimental levels enabling to predict optimum conditions for the said the objective. These techniques have been widely adopted for optimizing the processes of enzymes and peptides, solvents, polysaccharides, and other related molecules (Wang and Lu 2005). Three-dimensional (3D) graphs were generated from the response of pairwise combination of the four factors keeping the other two at their optimum level. The graphs are given here to highlight the roles played by various factors in the final yield of L-DOPA.

**Figure 1:**
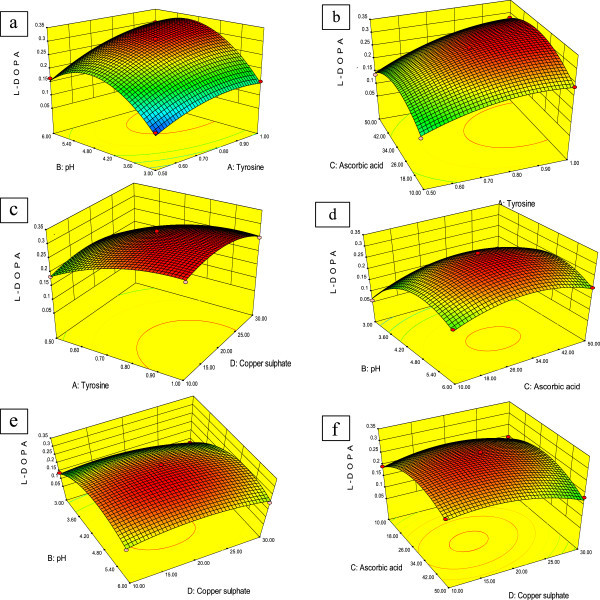
**Three-dimensional response surface curve showing the effect of interactions of (a) pH and L- tyrosine, (b) ascorbic acidand L-tyrosine, (c) L-tyrosine and CuSO4, (d) pH and ascorbic acid, (e) pHand CuSO4, (f) ascorbic acid and CuSO4 on L-DOPA production.**

Form the 3D and contour plot, it was clear that the effect of pH and tyrosine concentration on L-DOPA production was significant (Figure 1a) with significant interaction between these two factors. Tyrosine concentration showed the linear effect, whereas pH showed quadratic effect. L-DOPA yield increased with increase in tyrosine concentration, however the pH produced maximum yield at 4.2 to 5.8 pH range, above and below as the yield decreased. At lower pH, the increased tyrosine concentration had minimum effect as compared to higher pH. The enzyme responsible for this biotransformation is tyrosinase which has pH optima at pH 5.4 (Ali et al. 2006). The response surface curve is shown in Figure 1b, illustrating that the interaction between tyrosine and ascorbic acid moderately affected the yield of L-DOPA. The increase in tyrosine concentration provided increased substrate availability for biotransformation whereas, ascorbic acid prevents further conversion of the L-DOPA to dopaquinone and thus provides increased yield (Kim and Uyama 2005). Effect of the ascorbic acid was more evident at higher concentrations of tyrosine rather that lower concentration.

As evident from the Figure 1c and Table 2, tyrosine and copper sulphate had no significant interactions between them. Both the parameters showed the linear relationship causing increase in yield with increase in concentration. Tyrosinase being copper containing enzyme, production was increased in presence of copper sulphate (Claus and Decker 2006). The shape of the 3D response surface curve of the interaction between pH and ascorbic acid has been depicted in Figure 1d. Results indicated that L-DOPA production was drastically affected by a slight change in the levels of these two factors. The higher and lower concentrations of both factors resulted in lesser L-DOPA yield.

Figure 1e shows interactive effect of the pH and copper sulphate. The 3D response surface plot indicates that interaction of these components moderately affected the production of L-DOPA. The higher and lower levels of these components did not affect the L-DOPA yield drastically, but mid-levels provide a maximum yield. At optimum pH and Copper sulphate concentration the enzyme was efficiently active to produce maximum L-DOPA. Ascorbic acid and copper sulphate showed broad range of optimum concentration (Figure 1f). Both the parameters showed quadratic effect on L-DOPA production with optimum yield at center point above and below which, yield decreased.

### Validation of the experimental model

Validation was carried out under conditions predicted by the model. The optimized levels predicted by the model were tyrosine 0.894 g l^-1^, pH 4.99, ascorbic acid 31.62 mg l^-1^and copper sulphate 23.92 mg l^-1^. The predicted yield of L-DOPA with these concentrations was 0.319 g l^-1^, while the actual yield obtained was 0.309 g l^-1^. A close correlation between the experimental and predicted values was observed, which validates this model which signifies the RSM methodology over traditional optimization approach.

### Laboratory scale column reactor for L-DOPA production

Production of L-DOPA in a shake flask culture is limited by the fact that accumulated L-DOPA gets converted to DOPA quinine and subsequently to melanin. Hence, yield decreased over a period of time and product formed contained a mixture of L-DOPA and melanin. Column bioreactor was constructed keeping in mind that continuous supply of fresh medium and removal of formed product at the same rate will utilize the biomass potential at its maximum efficiency. Most influencing factor using reactor was flow rate and was optimized for L-DOPA production. Maximum L-DOPA production was obtained at 30 ml h^-1^ flow rate, whereas formation of melanin was predominantly observed at a flow rate of 15 ml h^-1^ Production rate was stable over a period of 48 h. In addition, antibiotic cefazolin was incorporated in biotransformation medium to avoid possible contamination and also to inhibit the diphenolase activity of the tyrosinase enzyme which would be beneficial to prevent further conversion of the L-DOPA to dopaquinone. Cefazolin inhibits diphenolase activity strongly than monophenolase activity (IC 50 for monophenolase is 7 mM whereas for diphenolase, IC 50 value is 0.21 mM) (Zhuang et al. 2009) and hence, was used as an antibiotic in biotransformation medium.

#### L-DOPA yield

**Figure 2:**
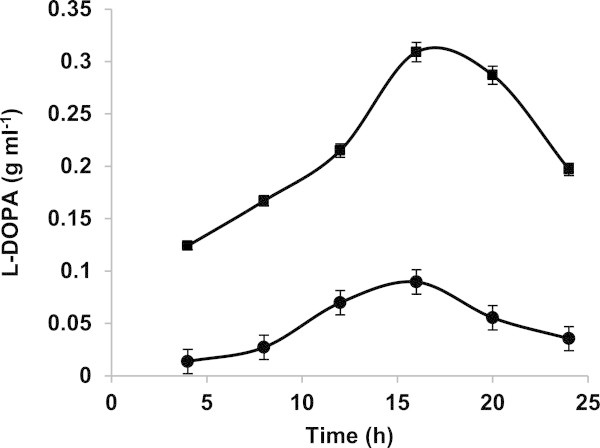
**L-DOPA yield using conditions before optimization and using the medium optimized by RSM.**  L-DOPA yield after optimization,  L-DOPA yield before optimization.

L-DOPA yields before and after optimization is depicted in Figure 2. Before optimization, the L-DOPA production started after 4 h with yield of 0.013 g l^-1^, increased gradually up to 16 h producing 0.0895 g l^-1^ L-DOPA and then decreased, after optimization, production started with yield of 0.124 g l^-1^, increased up to 0.309 g l^-1^ at 16 h and then decreased. Thus, optimization resulted in 3.45 fold increase in L-DOPA production. In case of column reactor, L-DOPA production was achieved starting from first hour with a stable yield of 0.2 g l^-1^. The decrease in the L-DOPA yield observed after the 16^th^ h in flask culture was attributed to the conversion of L-DOPA to further metabolites, such as dopaquinone and melanin (Ali et al., 2007; Inamdar et al. 2012).

Biosynthesis of L-DOPA was much better in terms of production rate as well as in time duration compared with most other reports (Table 3). The literature survey revealed that single and multiple stage cell suspension cultures of *M. pruriens* have been reported to produce L-DOPA within 15 and 30 days with a production rate of 0.025 and 0.39 mg l^-1^ h^-1^respectively (Chattopadhyay et al. 1994). *P. grandiflora* has produced L-DOPA at a rate 48.8 mg l^-1^ h^-1^ of in 16 h (Rani et al. 2007). In addition, previously reported L-DOPA production by bacterial sources mainly Bacillus sp. JPJ and Brevundimonas sp. SGJ exhibited higher production rate but used substances like pyrocatechol (toxic), activated charcoal and polyacrylamide gel (expensive), respectively, as enhancers (Surwase and Jadhav 2011; Surwase et al. 2012a). In case of continuous process using column reactor, L-DOPA production rate was 150 mg l^-1^ h^-1^ which is the maximum production rate ever reported using plant cultures. In addition, the optimized biotransformation medium was very simple devoid of large number of nutrients providing no interference on biotransformation, the ease of separation and also reducing the overall cost of production.

**Table 3:** **Comparison of L-DOPA production by biological methods**

Method	Rate of production (mg l^-1^ h^-1^)	Scale (ml)	References
***Mucuna pruriens*** **single-stage culture**	0.025	100	Chattopadhyay et al. ( 1994)
***Mucuna pruriens*** **two-stage culture**	0.39	100	Chattopadhyay et al. ( 1994)
***E. coli*** **cloned** ***E. herbicola*** **culture**	0.39	25	Foor et al. ( 1993)
**Immobilized tyrosinase on nylon 6,6**	1.7	500	Pialis et al. ( 1996)
**Immobilized tyrosinase-batch reactor**	1.7	500	Pialis et al. ( 1996)
***Aspergillus oryzae*** **(GCB-6)**	7.5	100	Ali et al. ( 2002)
**Immobilized tyrosinase batch reactor**	27.6	20	Vilanova et al. ( 1984)
***Portulaca*** **callus cultures**	48.8	100	Rani et al. ( 2007)
***Brevundimonas*** **sp. SGJ**	186.6	100	Surwase et al. ( 2012b)
***Pseudomonas sp. SSA***	180.6	100	Patil et al. ( 2013)
***Mucuna monosperma*** **flask culture**	19.31	50	Current study
***Mucuna monosperma*** **continuous culture**	200	Continuous culture	Current study

### Analysis of L-DOPA using HPTLC and HPLC

**Figure 3:**
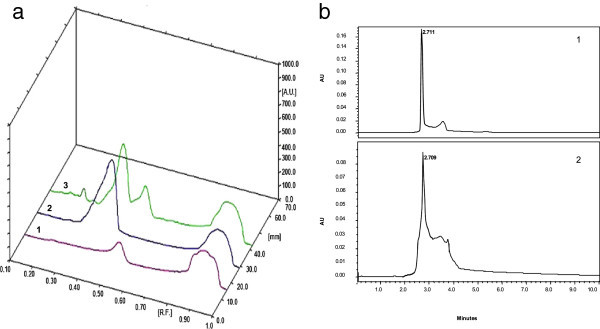
**Analysis of L-DOPA. (a)** HPTLC. 1. Standard tyrosine. 2. Standard L-DOPA. 3. Biotransformation product. **(b)** Analysis of L-DOPA using HPLC. 1. Standard L-DOPA. 2. Biotransformation product.

Presence of L-DOPA in the biotransformation product was confirmed by comparing with HPTLC and HPLC profile of the standard L-DOPA and product obtained after transformation. In HPTLC, standard L-DOPA showed major peak at Rf value 0.43 whereas, the transformation product also showed peaks at Rf value 0.43 (Figure 3a). So from the Rf values and the three dimensional profile of all the samples it was clear that test sample contained L-DOPA.

The HPLC elution profile of standard L-DOPA showed peak at the retention time 2.711 minutes, whereas, that of the transformation product was at retention time 2.709 minutes (Figure 3b). Thus HPLC analysis confirmed the presence of L-DOPA in the biotransformation product.

## Conclusion

Present study demonstrates the biotransformation potential of *M. monosperma* callus for L-DOPA production. Use of response surface methodology for optimization of media component is prerequisite for the large scale production which will increase the yield in significant amount with reduction in optimization time. Continuous production of L-DOPA was produced at a maximum production rate suggesting economy of the process. Thus, *M. monosperma* callus is a good alternative source for L-DOA production using biotransformation. Use of continuous culture will establish as a method of choice for L-DOPA production.

## Methods

### Tissue culture and callus initiation

Seed explants were washed well in 10% detergent, for 10 min before treating with 0.1% mercuric chloride for 15 min and were inoculated onto media containing different concentrations of hormones on full-strength MS medium (Murashige and Skoog 1962). Sucrose (3.0%) was used as the carbon source and 0.2% (w/v) Clarigel as solidifying agent and incubated at 25°C under a photo period of 12/12 h. NAA, 2,4-D and glutamine at different concentrations were used for callus initiation and proliferation. Optimum period for biomass production was determined. First sub culturing was done after 24 h and then after every 20–25 days.

### Biotransformation of L-tyrosine to L-DOPA

Potential of biotransformation of L-tyrosine to L-DOPA was exploited in 50 ml citrate phosphate buffer (0.1 M, pH 5.0) containing 1.0 g l^-1^ tyrosine in presence of different activators such as CuSO_4_, ascorbic acid. Reaction mixture was incubated at 120 rpm on rotary shaker for 14 h and L-DOPA produced was analyzed by Arnow’s method (Arnow 1937).

### Experimental design

Based on the results obtained in previous experiments and literature survey, concentrations of tyrosine, CuSO_4_, ascorbic acid and pH were found to affect L-DOPA production significantly. Hence, these variables were optimized using Box-Behnken design for maximum biotransformation. Four variables at three levels were used to fit a polynomial model (Box and Behnken 1960) and the boundary conditions for each parameter are as depicted in Table 4. The Design Expert software (version 8.0, Stat-Ease Inc., Minneapolis, USA) was used in the experimental design and data analysis. A quadratic model is designed such that the variance of Y is constant for all points equidistant from the center of the design. Response surface graphs were obtained to understand the effect of the variables, individually and in combination, and to determine their optimum levels for maximum L-DOPA production. All trials were performed in triplicate, and the average yield was used as response Y. The significance of the model equation and model terms was evaluated by ‘P’ value and *F-*test. The quality of the quadratic model equation was expressed by determination coefficient *R*^2^ and adjusted *R*^2^. Analysis of variance (ANOVA) was applied to evaluate the statistical significance of the model. Model fitting was confirmed with ‘Lack of Fit’ test. Adequacy of the predicted model was evaluated with Normal probability plot and Box-Cox analysis. The optimal values were obtained by solving the regression equation and analyzing 3-dimensional response surface plots and contour plots.

**Table 4:** **Level and range of independent variables chosen for L-DOPA production**

Factor	Variable	Unit	Range and level of coded values		
			−1	0	+1
A	L-Tyrosine	g l^-1^	0.5	0.75	1.0
B	pH	Unit	3	4.5	6
C	Ascorbic acid	mg l^-1^	10	30	50
D	Copper sulphate	mg l^-1^	10	20	30

### Laboratory scale column reactor for L-DOPA production

**Figure 4:**
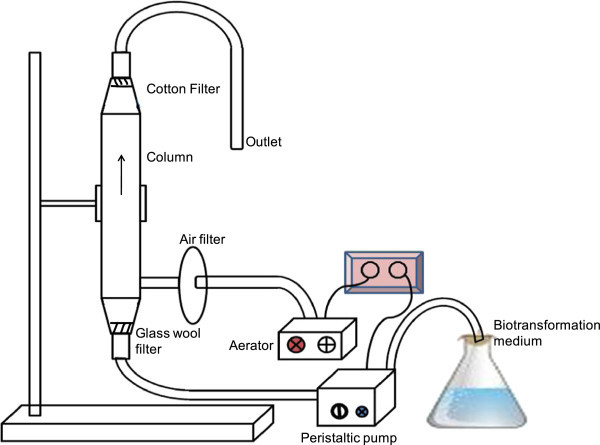
**Schematic diagram of laboratory scale column reactor.**

Continuous production of L-DOPA was achieved using a laboratory scale column (3 cm Φ × 30 cm) with a provision for aeration at the bottom. Medium was allowed to pass through bottom of the column using peristaltic pump and produced L-DOPA was recovered from the top of the column (Figure 4). Sterile air was introduced in to the column using aerator and bacteria proof filter. Flow rate was controlled using peristaltic pump.

### L-DOPA assay

L-DOPA produced in the broth was determined according to Arnow’s method (Arnow 1937). The reaction mixture was centrifuged at 5000 r.p.m. for 15 min., and 1 ml supernatant was added with 1 ml of 0.5 N HCl, 1 ml of nitrite molybdate reagent, and 1 ml of 1 N NaOH. The absorbance was measured at 460 nm using a double-beam UV-visible spectrophotometer (Shimadzu, Japan).

### Analysis of L-DOPA using HPTLC and HPLC

High performance thin layer chromatography (HPTLC) analysis was performed by using HPTLC system (CAMAG, Switzerland). The 10 μl of the standard L-DOPA and biotransformation product were loaded on pre-coated HPTLC plates (Silica gel 60 F 254, Merck, Germany), by using spray gas nitrogen and TLC sample loading instrument (CAMAG LINOMAT 5). The HPTLC plates were developed in solvent system *n*-butanol: acetic acid: water; 4:1:1 in a CAMAG glass twin-through chamber (10 × 10 cm) previously saturated with the solvent for 30 min (Inamdar et al, 2012). After development, the plate was observed in UV chamber and scanned at 282 nm with slit dimension 5 × 0.45 mm by using TLC scanner. The results were analyzed by using HPTLC software WinCATS 1.4.4.6337.

High performance liquid chromatography (HPLC) analysis was carried out (Waters model no. 2690) on C 8 column (symmetry, 4.6 mm × 250 mm) by using methanol as a mobile phase with a flow rate of 1 ml min^-1^ for 10 min and UV detector at 280 nm. The standard L-DOPA and biotransformation product were prepared in HPLC grade water and used as samples and 10 μl of each sample was injected in HPLC column.

## Abbreviations

2: 4 D: 2,4-Dichlorophenoxyacetic acid
HPLC: High performance liquid chromatography
HPTLC: High performance thin layer chromatography
L-DOPA: 3,4-dihydroxyphenyl-L-alanine
NAA: Naphthalene acetic acid
PD: Parkinson’s disease
RSM: Response surface methodology.
